# A high resolution melting method for the molecular identification of the potentially toxic diatom *Pseudo-nitzschia* spp. in the Mediterranean Sea

**DOI:** 10.1038/s41598-017-04245-z

**Published:** 2017-06-26

**Authors:** Laura Pugliese, Silvia Casabianca, Federico Perini, Francesca Andreoni, Antonella Penna

**Affiliations:** 1 Department of Biomolecular Sciences, University of Urbino, Viale Trieste 296, 61121 Pesaro, Italy; 2 Conisma, Consorzio Interuniversitario per le Scienze del Mare, Pz. Flaminio 9, 00196 Rome, Italy; 3 CNR–Institute of Marine Sciences (ISMAR), Largo Fiera della Pesca, 60125 Ancona, Italy

## Abstract

The aim of this study was to develop and validate a high resolution melting (HRM) method for the rapid, accurate identification of the various harmful diatom *Pseudo-nitzschia* species in marine environments. *Pseudo-nitzschia* has a worldwide distribution and some species are toxic, producing the potent domoic acid toxin, which poses a threat to both human and animal health. Hence, it is important to identify toxic *Pseudo-nitzschia* species. A pair of primers targeting the LSU rDNA of the genus *Pseudo-nitzschia* was designed for the development of the assay and its specificity was validated using 22 control DNAs of the *P. calliantha*, *P. delicatissima/P. arenysensis* complex and *P. pungens*. The post-PCR HRM assay was applied to numerous unidentified *Pseudo-nitzschia* strains isolated from the northwestern Adriatic Sea (Mediterranean Sea), and it was able to detect and discriminate three distinct *Pseudo-nitzschia* taxa from unidentified samples. Moreover, the species-specific identification of *Pseudo-nitzschia* isolates by the HRM assay was consistent with phylogenetic analyses. The HRM assay was specific, robust and rapid when applied to high numbers of cultured samples in order to taxonomically identify *Pseudo-nitzschia* isolates recovered from environmental samples.

## Introduction

Diatoms (class Bacillariophyceae) are among the most productive eukaryotic microalgae. They are found in oceans around the world and play a fundamental role in global biogeochemical cycles^1^. Within the Bacillariophyceae class, the genus *Pseudo-nitzschia* is found in polar, temperate, subtropical and tropical regions^2^, although some *Pseudo-nitzschia* species are limited to distinct regional areas. Among the more than 37 species identified worlwide^3^, at least 12 *Pseudo-nitzschia* species are toxic. These species produce domoic acid (DA), a neurotoxin causing amnesic shellfish poisoning (ASP), which is responsible for many toxic blooms worldwide^2^. Blooms of *Pseudo-nitzschia* spp. can be stimulated by nutrient (mainly nitrate and phosphate) input from various sources, such as upwelling, turbulence or riverine inputs, deriving largely from anthropogenic nutrient loads of agricultural and sewage origins^4^. However, *Pseudo-nitzschia* blooms or occurrences are often a seasonal phenomenon in many coastal sites, and the frequency of toxic blooms generated by several species of *Pseudo-nitzschia* is increasing in various coastal areas worldwide^2^. The impact of DA is evident in the marine food web. In fact, DA has been detected in the tissues of many invertebrates, fish, birds and marine mammals^5–7^. In Canada in 1987 consumption of contaminated seafood^8^ caused a serious outbreak of ASP resulting in human mortality. However, since this dramatic episode, there have been no reports of human deaths caused by ASP thanks to the institution of effective monitoring programs. Nevertheless, DA occurrence can have negative economic impact on shellfish aquaculture farms, since the molluscan shellfish remain the only known vector of DA to humans.

The regions that are most affected by DA are Northern Europe, Canada and Northern United States. Low levels of DA have been detected in the Mediterranean Sea, but its presence has not been associated with harmful blooms. However, various *Pseudo-nitzschia* species, such as *P. brasiliana*, *P. calliantha*, *P. galaxiae*, *P. multistriata* and *P. pseudodelicatissima* have been found toxic^9–13^. Furthermore, today, several species of *Pseudo-nitzschia* pose a contamination risk along the coast of North Africa. The northern Adriatic Sea (Mediterranean Sea) is a high mesotrophic area strongly influenced by inputs from the Po River, which sustains blooms dominated by numerous frequently occurring diatom species with considerable biomass, including various *Pseudo-nitzschia* spp. with an abundance range of 10^4^–10^5^ cells l^−1^ ^14–16^. The most frequently occurring species are *P. delicatissima* complex, *P. seriata* complex, *P. calliantha*, *P. pungens*, *P. fraudulenta* ^17, 18^, and toxic blooms have recently been linked to ASP toxin or DA accumulation in Adriatic shellfish^19–21^. However, in the NW Adriatic Sea, this genus is more widespread than other potentially harmful phytoplankton taxa, therefore, it is important to check the DA levels associated to *Pseudo-nitzschia* species blooms or occurrences.

Multiple toxigenic *Pseudo-nitzschia* species frequently coexist in the same environment, even during bloom events that appear to be dominated by a single species^8^. Since the genus *Pseudo-nitzschia* includes a large number of species, their accurate taxonomical identification is important because they can be associated with domoic acid production^3^. To date, species identification or description has often been performed by integrating different methodological approaches based on scanning and transmission electron microscopy, and molecular analyses. However, the light microscopy does not always provide the resolution required for the identification of various *Pseudo-nitzschia* species^22, 23^. Further, despite concerted efforts, the taxonomy of *Pseudo-nitzschia* has still being updated, and new morphological species complexes and/or cryptic and pseudo-cryptic species (i.e. *P. delicatissima* or *P. pseudodelicatissima* complex) have recently been described within the genus^24, 25^. Molecular taxonomy studies based on different genetic markers, including ribosomal RNA gene (LSU) and internal transcribed spacers (ITS regions), cytochrome oxidase 1 (cox 1) and chloroplast genes of ribulose 1,5 biphosphate carboxylase (rbcl), have uncovered numerous cases of genetically distinct, and at times reproductively isolated, groups of strains or genetic lineages that could not be easily distinguished with light microscopy^26–28^.

Recent molecular approaches, such as qPCR^13, 29, 30^, ARISA^31^, microarray^32–34^ and dot blot hybridization systems^35^ have been used for specific and sensitive *Pseudo-nitzschia* species identification and/or quantification from clonal cultures and field samples^36^. These methods are essentially based on the evaluation of the sequence variation and design of oligonucleotide primers and/or probes in target nucleotide regions and they allow the accurate identification of various species. Among the molecular techniques used to analyze small genetic mutations, such as single nucleotide polymorphisms (SNPs), we find the post PCR high resolution melting (HRM) curve analysis. This technique is based on the melting properties of double-stranded DNA (dsDNA). Different melting profiles are obtained from the transition of dsDNA to denaturated single-stranded DNA (ssDNA) as a result of a gradual temperature increase after PCR amplification. Both processes, PCR and gradual denaturation, take place in the same tube during a real-time run lasting less than two hours. The recent development of HRM was made possible by a new generation of dyes designed for this technique and technological improvements in real-time PCR instruments. The HRM method has mainly been used for genotyping microrganisms^37–40^ and it is considered the simplest method for genotyping and detecting mutations since it can be performed immediately after qPCR. This post PCR HRM assay can provide higher throughput and specificity at a lower cost and with reduced analysis time of the cultured sample examination compared to qPCR, microarray or rDNA sequencing. In fact, a single HRM reaction can process many samples simultaneously. Moreover, the melting HRM profiles seem to be more specific than some probe signals, and because of the high throughput analysis, the HRM assay has lower costs than PCR and sequencing reactions.

In this study, the first molecular assay based on HRM curve analysis was developed and applied to detect three different potentially toxic *Pseudo-nitzschia* species, such as *P. calliantha*, *P. delicatissima* complex and *P. pungens*, which are very common in the NW Adriatic Sea. These species are always retrieved in blooming events and/or occurrences of mixed diatom species^18^ in this area of the Mediterranean where aquaculture farming is widespread. Hence, these species pose a potential risk of the DA accumulation in molluscan shellfish, although to date, DA levels of *P. delicatissima* complex are low^13^.

Several cultured strains of *Pseudo-nitzschia* spp., isolated from seawater samples, were analyzed by the post PCR HRM assay, which yielded precise accurate identification of three distinct species of *Pseudo-nitzschia* based on the different melting curve profiles that were generated. The HRM-based identifications were also confirmed by phylogenetic analyses based on both LSU and ITS rDNA sequences.

This newly developed method proved to be specific, accurate and rapid in discriminating numerous cultured isolates of the *Pseudo-nitzschia* species or complex, which are very difficult to identify using traditional light microscopy. Knowledge of the diverse composition of potentially toxic *Pseudo-nitzschia* spp. can provide information on the presence and frequency of target species in regional coastal waters in the NW Adriatic Sea. Such information can be important for the management of harmful algal blooms occurring in areas where molluscan shellfish farms are widespread.

## Methods

### Sampling, isolation and cultures

The sampling sites were located 3000 m off the Italian coast (northwestern Adriatic Sea) at Tavollo (43°59′.30 N; 12°46′.42 E), Foglia (43°56′.55 N; 12°56′.18E) and Metauro (43°50′.54 N; 13°05′.9 E) river transects. Several strains of *P. calliantha*, *P. delicatissima*, *P*. cf. *arenysensis* and *P. pungens* isolated 500 m off the coast of Pesaro in 2009, 2010 and 2013, as described in Penna *et al*.^13^, were used for the HRM assay development (Table S1). An additional 29 *Pseudo-nitzschia* strains were isolated from net samples using single cell isolation technique during the period from November 2014 to March 2015 (see Results section). All isolates were maintained in f/2 medium^41^ at 16 ± 1 °C on a 12:12 h light:dark cycle, at an irradiance of 100 µmoL photons m^−2^ s^−1^.

Light microscopy (LM) observations of living cells were carried out using an Axiovert 40 CFL, Zeiss at 200x and 400x magnifications.

### Genomic DNA extraction

Exponential phase cultures of *Pseudo-nitzschia* spp. were harvested by centrifugation at 4,000xg for 20 min at room temperature. Total genomic DNA was extracted from pellets using the DNeasy Plant Kit (Qiagen, Valencia, CA, USA) according to the manufacturer’s instructions. DNA integrity was assessed by electrophoresis on agarose gel (0.8% w/v) and visualized by standard ethidium bromide staining under UV light. Quantification was performed using a Qubit fluorometer with a Quant-iT dsDNA HS Assay Kit (Invitrogen, Carlsbard, CA, USA).

### HRM primer design and specificity

**Figure 1:**
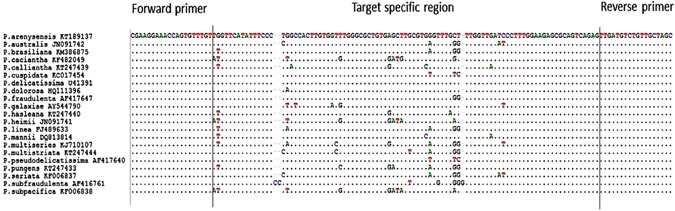
The alignment of consensus LSU rDNA sequences of 21 *Pseudo-nitzschia* species. The sequences were aligned using the Bioedit Sequence Alignment v. 7.0.5.3. The common forward and reverse primers flanked species-specific regions of the 21 *Pseudo-nitzschia* species considered under investigation. The target LSU rDNA amplified region was 130–133 bp in length and located in the 384 to 514 nucleotide position.

A total of 21 LSU rDNA sequences of various *Pseudo-nitzschia* species mainly present in the Mediterranean Sea (Fig. 1)^25, 42, 43^, available in GenBank, were aligned using the BioEdit Sequence Alignment Editor v. 7.0.5.3^44^, to check for a SNP-carrying region flanked by highly conserved sequences suitable for primer design. The primers were designed using Primer-BLAST^45^. The primers for the amplification of the 130 − 133 bp fragment, which was positioned from the 384 to 514 nucleotide position, were HRM_PSEUDO_F forward (5′-GCGAAGGAAACCAGTGTTTGT-3′) and HRM_PSEUDO_R reverse (5′-GCTAGCAACAACAGACATCAACTCT-3′). The primers were synthesized by Eurofins MWG Operon (Ebersberg, Germany). The *Pseudo-nitzschia* primer specificity was examined *in silico* using BLAST and tested in qPCR with both target and various purified genomic DNAs of *Pseudo-nitzschia* spp. including *P. calliantha*, *P. delicatissima*, *P*. cf. *arenysensis*, *P. pungens*, *P. fraudulenta*, *P. multistriata* and *P. pseudodelicatissima*, which are common in the northwestern Adriatic Sea. The specificity of the assay was also evaluated using the DNA of other 23 phytoplankton taxa comprising other 6 diatom species, 14 dinoflagellate species and 3 species of Raphidophyceae. The PCR protocol is described below. The melting curve analysis was performed to check primer dimers and PCR products from misannealed primers. The PCR products were electrophoresed and analyzed on 1.8% (w/v) agarose gel. The amplified PCR fragments were sequenced by GATC Biotech AG, Köln, Germany to confirm the target specific DNA fragment. Each PCR reaction testing the specificity of the assay was performed in duplicate.

### qPCR HRM assay

The qPCR HRM assay was performed in a final volume of 25 μl containing primers at final concentrations of 500 nM and 200 µM of each GeneAmp dNTP; 1.5 mM MgCl_2_; 1X Reaction AmpliTaq Gold 360 Buffer; 1X Reaction MeltDoctor HRM Dye, (MeltDoctor™ HRM Dye, a stabilized form of the fluorescent SYTO® 9 double-stranded nucleic acid stain), 1.25 U of AmpliTaq Gold 360 DNA Polymerase (Applied Biosystems, Foster City, CA, USA), and 1 ng of DNA template. PCR was carried out using the StepOne Real-Time PCR System (Applied Biosystems, Foster City, CA, USA). The thermal cycling conditions consisted of 10 min at 95 °C, followed by 40 cycles at 95 °C for 10 s and 60 °C for 1 min. HRM assay was performed from 60 °C to 95 °C with a ramp rate of 0.3%. All PCR experiments were performed in duplicate including target positive controls of *P. calliantha* CBA72, *P. pungens* CBA100 and non-template controls (NTC). As *P. delicatissima* and *P. arenysensis* shared the same LSU rRNA target amplicon, the *P. delicatissima* CBA144 was used as a control for the *P. delicatissima/P. arenysensis* phylogenetic complex.

The raw melting curve data were processed by the High Resolution Melt Software v. 3.0.1 (Applied Biosystems, Foster City, CA, USA). The pre- and post-melt regions were set as close as possible to the melting transition region. Positive controls, one for each species tested, were set in the HRM software assigning each control to its corresponding well. Samples were analyzed and the software automatically made a call for each sample according to the shape of the melt curves aligned to the controls and the melting temperature (T_m_).

### HRM assay validation and application for *Pseudo-nitzschia* species identification

The method was validated using several DNAs (n = 22) of *Pseudo-nitzschia*, already used as controls (as above), such as the target species of the *P. calliantha*, *P. pungens* and *P. delicatissima*/*P*. cf. *arenysensis* complex. In particular, 7 strains of each species, as *P. calliantha*, *P. pungens* and *P. delicatissima*, and 1 strain of *P*. cf. *arenysensis*, were used (Table S1). The method was subsequently applied to 29 strains of unidentified *Pseudo-nitzschia* spp. isolated from seawater off the coast (northwestern Adriatic Sea) between November 2014 and March 2015, as previously described. The HRM assay was applied for taxon-specific identification.

### Statistical analyses

HRM data analysis was performed with the Kruskall-Wallis and Mann-Whitney tests to determine whether there were significant differences in the average Tm values among and between the *Pseudo-nitzschia* spp. isolates. All statistical calculations were performed using PAST ver. 2.17 with a p < 0.05 determining significance.

### Molecular and phylogenetic analyses

Representative strains of *Pseudo-nitzschia* spp. identified by the HRM assay were analyzed to confirm the species-specific taxonomical assignment by LSU and ITS-5.8S rDNA sequence alignment and phylogenetic analyses. The sequences of ribosomal genes obtained from new *Pseudo-nitzschia* cultured isolates were deposited in EMBL-EBI-ENA. Other ribosomal sequences of *Pseudo-nitzschia* spp. isolates were included in this study. All sequences are listed in Table S2. The LSU rDNA was amplified and sequenced using D1R or D2C primers targeting the D1-D2 region of the nuclear LSU rDNA^46^. The ITS region of the rDNA was amplified and sequenced using the universal primer ITSA and ITSB^47^ or ITS1R and ITS1F^48^. The PCR reaction for the LSU rDNA was as follows: tubes contained 50 µl of mixture of 200 µM of dNTPs; 0.4 µM of each primer, 4 mM of MgCl_2_, 1X reaction buffer (Master TaqBuffer, 5 PRIME, Germany), 1U Taq DNA Polymerase (5 PRIME, Germany) and 0.5-1 ng DNA template. PCR thermal cycling conditions were as follows: an initial denaturation at 95 °C for 10 min, 35 cycles of 1 min at 95 °C, 1 min at 50 °C, and 2.5 min at 72 °C and a final extension step of 7 min at 72 °C. The PCR reaction for the ITS-5.8S rDNA using ITSA and ITSB primers was as follows: tubes contained 50 µl of mixture of 200 µM of dNTPs; 0.2 µM of each primer, 1 mM of MgCl_2_, 0.75X TaqMasterPCR Enhancer (5 PRIME, Germany), 1X reaction buffer (Master TaqBuffer, 5 PRIME, Germany), 1U Taq DNA Polymerase (5 PRIME, Germany) and 0.5–1 ng DNA template. The PCR using ITS1R and ITS1F primers was carried out in a mixture as described above, with the following exceptions: 0.4 µM of each primer, 4 mM of MgCl_2_ and 0.5X TaqMasterPCR Enhancer (5 PRIME, Germany). PCR thermal cycling conditions were as follows: an initial denaturation at 95 °C for 10 min, 35 cycles of 30 s at 94 °C, 30 s at 55 °C min or 50 °C, and 30 s at 72 °C and a final extension step of 10 min or 2 min at 72 °C.

All PCR amplified products were purified using the MinElute Gel Extraction Kit (Qiagen, Valencia, CA, USA), and the products were directly sequenced with the ABI PRISM BigDye Terminator Cycle Sequencing Kit v. 1.1 on the ABI 310 Genetic Analyzer (Applied Biosystem, Foster City, CA, USA). Standard thermal cycling conditions were used for both templates setting the annealing temperature according to the template (60 °C and 50 °C for ITS and LSU PCR specific primers, respectively). Difficult templates and repeated regions were solved increasing initial denaturation time and modifying thermal cycling condition as follows: denaturation at 96 °C for 10 sec and annealing/extension at 50 °C for 40 cycles.

The LSU and ITS-5.8S sequences were aligned using MAFFT software. Short aligned sequences and ambiguously aligned positions were excluded from the alignment manually or using Gblocks (http://molevol.cmima.csic.es/castresana/Gblocks.html) with default settings. The neighbor-joining (NJ), maximum parsimony (MP) and maximum likelihood (ML) analyses were performed in MEGA v. 6.06^49^. The robustness of the NJ, MP and ML trees was tested by bootstrapping using 1000 pseudo-replicates. Distance and maximum likelihood trees were built based on the substitution model selected through the Akaike Information Criterion option implemented in MEGA v. 6.06. The most appropriate evolutionary models for LSU and ITS-5.8S gene rDNA alignment were found to be HKY + G and HKY + G + I, respectively. The MP analyses were performed using the Tree-Bisection-Redrafting (TBR) algorithm with search level 1, in which the initial trees were obtained by the random addition of sequences (10 replicates). All positions containing gaps and missing data were eliminated. Bayesian analyses were performed using MrBayes 3.2.3^50^ with the following settings: four Markov chains were run for 2,000,000 generations with a sampling frequency of 100 generations. Log-likelihood values for sampled trees were stabilized after almost 200,000 generations. The last 18,000 trees were used to estimate Bayesian posterior probabilities, whereas the first 2,001 were discarded as burn-ins. Results from two-independent runs were used to construct a majority-rule consensus tree containing the posterior probabilities.

The sequences of *Fragilariopsis rhombica* 5–17 AF7656 and *Fragilariopsis* sp. NL2010 GU170665 were used as an outgroup for the *Pseudo-nitzschia* LSU and ITS-5.8S gene phylogenetic analyses, respectively.

## Results

### HRM primer design and specificity

The *Pseudo-nitzschia* spp. primers, designed to amplify the target sequence of the LSU rDNA region, were examined *in silico* using BLAST and they were found to be specific to *P. calliantha*, *P. delicatissima*, *P*. cf. *arenysensis* and *P. pungens*. No non-specific products were detected, and amplification was not obtained in any template controls (NTCs). According to the *in silico* analysis, the PCR product size was 130 bp. The specificity of the qPCR assay was also tested by using DNA from other *Pseudo-nitzschia* and microalgal species. Negative amplifications were obtained. All these results showed that the HRM assay was highly specific for targeting the *Pseudo-nitzschia* species.

### Validation of the qPCR HRM assay

The HRM assay proved able to successfully distinguish *Pseudo-nitzschia* species or complex such as *P. calliantha*, *P. pungens* and *P. delicatissima/P*. cf. *arenysensis*. All Ct values ranged from 20 to 24. The melting curve variation of the *Pseudo-nitzschia* spp. can be plotted in various ways, including aligned and difference plots, according to the melting behavior of their amplicons determined using the High Resolution Melt software ver. 3.0.1 (Applied Biosystems) (Figure S1). As shown in the plots, the melting curves of *Pseudo-nitzschia* spp. can be distinctly separated by both the silhouettes of the curves and the Tm for each species or complex. Three different average Tm values of 84.56 ± 0.18; 85.22 ± 0.06; 85.05 ± 0.09 were assigned to *P. calliantha*, *P. delicatissima/P*. cf. *arenysensis* and *P. pungens*, respectively. The Tm values were highly reproducible across 22 repeated melt curve runs (7 and 8 melt curve runs for *P. calliantha* and *P. pungens* and *P. delicatissima/P*. cf. *arenysensis*, respectively). The Kruskall Wallis test demonstrated that the three Tm values were significantly different (Hc = 22.32, p < 0.001). Moreover, the *a posteriori* pairwise Mann-Whitney test showed that the differences between *Pseudo-nitzschia* species were significant with the Bonferroni correction (p < 0.001).

### Analysis of *Pseudo-nitzschia* spp. isolates

**Figure 2:**
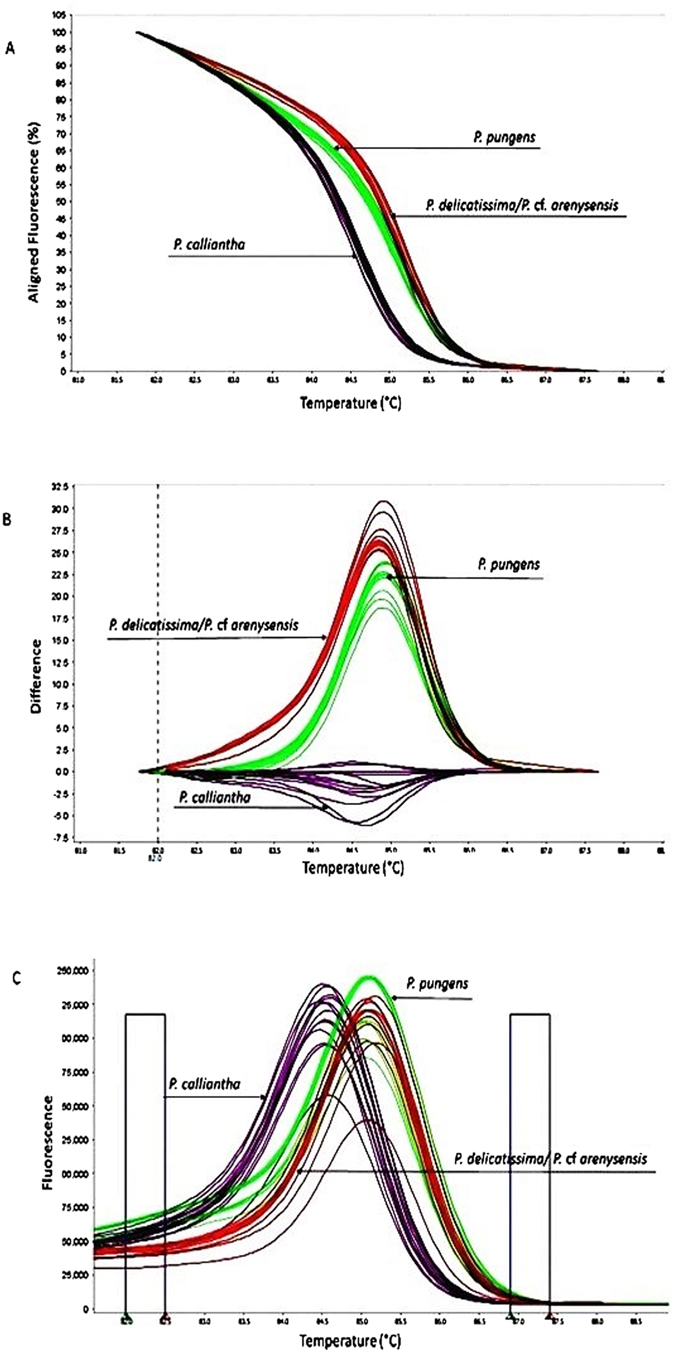
Melting curve variance of the *P. calliantha* (n = 12 strains), *P. delicatissima/P*. cf. *arenysensis* (n = 10 strains) and *P. pungens* (n = 7 strains) in **(A)** aligned, **(B**) difference and **(C**) derivative plot analyses; from left to right, vertical bars represent the pre and post – melt regions. A melting curve generated by a DNA positive control for each taxon was also included. Only one replicate of the HRM assay experiment for each strain is shown.

The method was subsequently applied for the analysis of unknown isolates of the *Pseudo-nitzschia* spp., collected between November 2014 and March 2015 in the northwestern Adriatic Sea. A total of 29 *Pseudo-nitzschia* spp. isolates were analyzed using the qPCR HRM assay. The melting profiles generated by PCR products of unidentified isolates were evaluated, and it was observed that the *Pseudo-nitzschia* spp. in the unknown strains showed consistency in their corresponding Tm values and curve silhouettes, which were similar to those generated by DNA positive controls. In the end, the melting profile from the isolates could be clustered into three groups using the auto-calling mode of the High Resolution Melt software (Applied Biosystems), and identification of the *Pseudo-nitzschia* spp. was made by comparing their silhouettes and Tm values to those of controls. The confidence interval for auto-called results ranged between 96–100% (Fig. 2). A total of 12 strains of *P. calliantha*, 10 strains of *P. delicatissima/P*. cf. *arenysensis*, and 7 strains of *P. pungens* were identified (Table 1).

**Table 1:** List of *Pseudo-nitzschia* spp.

Strain	Species identified by HRM	Collection site	Sampling date
CBA 172	*P. calliantha*	Tavollo	20 November 2014
CBA 173	*P. calliantha*	Tavollo	20 November 2014
CBA 181	*P. calliantha*	Tavollo	19 March 2015
CBA 183	*P. calliantha*	Tavollo	19 March 2015
CBA 189	*P. calliantha*	Tavollo	19 March 2015
CBA 191	*P. calliantha*	Metauro	20 March 2015
CBA 192	*P. calliantha*	Metauro	20 March 2015
CBA 193	*P. calliantha*	Tavollo	20 March 2015
CBA 194	*P. calliantha*	Metauro	20 March 2015
CBA 198	*P. calliantha*	Tavollo	13 January 2015
CBA 203	*P. calliantha*	Tavollo	13 January 2015
CBA 205	*P. calliantha*	Tavollo	13 January 2015
CBA 159	*P. delicatissima/P*. cf. *arenysensis*	Tavollo	20 November 2014
CBA 161	*P. delicatissima/P*. cf. *arenysensis*	Tavollo	20 November 2014
CBA 163	*P. delicatissima/P*. cf. *arenysensis*	Tavollo	20 November 2014
CBA 165	*P. delicatissima/P*. cf. *arenysensis*	Tavollo	20 November 2014
CBA 166	*P. delicatissima/P*. cf. *arenysensis*	Tavollo	20 November 2014
CBA 167	*P. delicatissima/P*. cf. *arenysensis*	Tavollo	13 January 2015
CBA 168	*P. delicatissima/P*. cf. *arenysensis*	Tavollo	13 January 2015
CBA 169	*P. delicatissima/P*. cf. *arenysensis*	Tavollo	13 January 2015
CBA 170	*P. delicatissima/P*. cf. *arenysensis*	Tavollo	13 January 2015
CBA 171	*P. delicatissima/P*. cf. *arenysensis*	Tavollo	13 January 2015
CBA 179	*P. pungens*	Metauro	20 March 2015
CBA 180	*P. pungens*	Tavollo	20 March 2015
CBA 182	*P. pungens*	Tavollo	19 March 2015
CBA 184	*P. pungens*	Tavollo	19 March 2015
CBA 186	*P. pungens*	Tavollo	19 March 2015
CBA 2S	*P. pungens*	Foglia	10 February 2015
CBA 3S	*P. pungens*	Foglia	10 February 2015

strains isolated from the NW Adriatic Sea 3000 m off the coast. The strains were analyzed and subsequently identified using the HRM assay.

**Figure 3:**
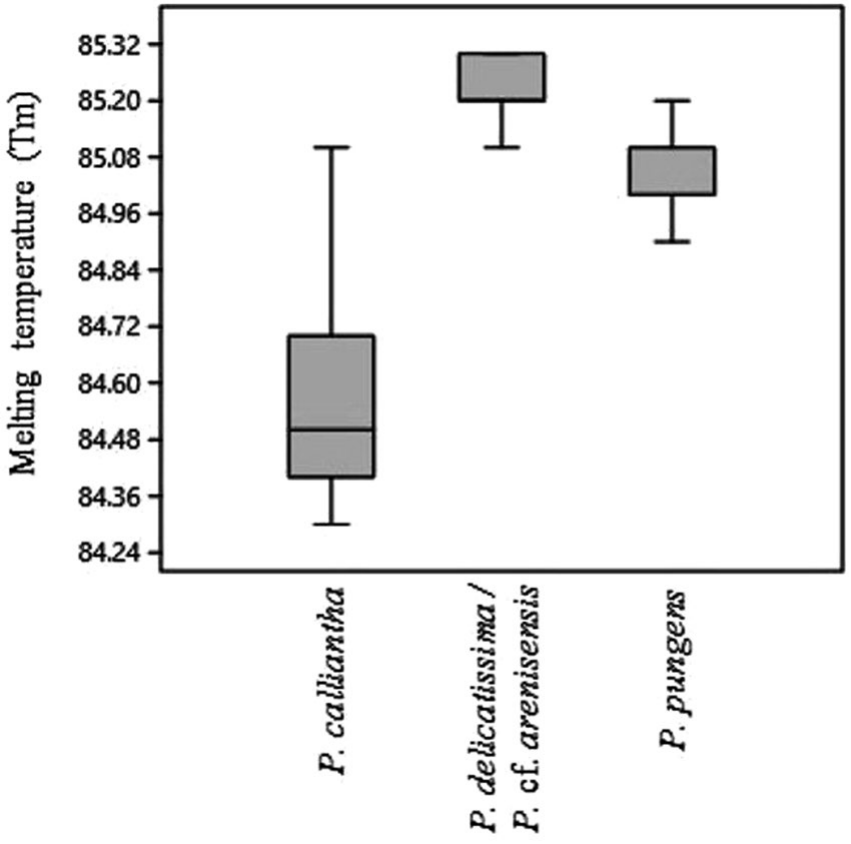
Box plot showing melting curve temperatures (Tm) of *Pseudo-nitzschia* spp. isolates collected in the NW Adriatic Sea and used in this study. The Kruskall Wallis test shows significant differences in average Tm values among species (p < 0.001).

All melting curve peak Tm values, which included values of controls and isolates for each taxon, namely *P. calliantha* (n = 19), *P. delicatissima/P*. cf. *arenysensis* (n = 18) and *P. pungens* (n = 14), are illustrated in the box plot (Fig. 3). The Tm values among the three taxa were found to be significantly different using the Kruskal-Wallis test (Hc = 60.4, p < 0.001). Furthermore, a *posteriori* pairwise Mann-Whitney comparisons showed that there was a significant difference between *P. calliantha*, *P. delicatissima/P*. cf. *arenysensis*, and *P. pungens* (p < 0.001 after the Bonferroni correction).

*Pseudo-nitzschia* spp. strains identified by the HRM assay were then verified by phylogenetic analyses based on LSU and ITS-5.8S rDNA sequences. All the strains analyzed using the HRM assay were sequenced and included in the phylogenetic analyses. Only, representative strains of the three NW Adriatic *Pseudo-nitzschia* spp. are shown.

### Phylogenetic analyses of *Pseudo-nitzschia* spp. LSU and ITS-5.8S ribosomal genes

The final alignments of *Pseudo-nitzschia* spp. ribosomal gene sequences, as namely LSU and ITS-5.8S, with *Fragilariopsis* as an outgroup, were as follows: LSU was 529 bp in length (A = 25.8%, T = 27.1%, C = 17.2%, G = 30%) with 515 total informative sites, excluding gaps, and 99 polymorphic sites, of which 65 were parsimony sites. ITS-5.8S was 890 bp in length (A = 27%, T = 34.8%, C = 18.2%, G = 20%) with 468 total informative sites excluding gaps and 218 polymorphic sites, of which 245 were parsimony sites.

Based on single LSU and ITS-5.8S rDNA sequences, only minor differences between the NJ, MP, ML and Bayesian inference analyses were found; therefore, only the ML phylogenetic trees are presented. The LSU rDNA phylogeny that was obtained from 40 isolates of the *Pseudo-nitzschia* spp. showed that NW Adriatic representative strains, identified as *P. pungens* (CBA179 and CBA180) and *P. calliantha* (CBA192 and CBA194) by the HRM assay, clustered in the clades of the corresponding species. By contrast, Adriatic strains identified as *P. delicatissima/P*. cf. *arenysensis* by the HRM assay and sharing identical LSU sequences, grouped together in a well-supported clade of *P. arenysensis*, as a sister to a clade including *P. delicatissima/P. micropora/P. dolorosa* within the *P. delicatissima* complex (Figure S2). Only four representative strains (CBA159, CBA165, CBA163, CBA169) of Adriatic *P*. cf. *arenysensis* were shown. All these lineages were strongly supported by high bootstrap and posterior probability values.

The ITS-5.8S rDNA phylogeny that was obtained from 31 isolates of the *Pseudo-nitzschia* spp. showed similar tree topology to the LSU rDNA phylogeny, confirming that the NW Adriatic representative strains, identified as *P. pungens* (CBA179 and CBA180) and *P. calliantha* (CBA193 and CBA194) by the HRM assay grouped with these corresponding taxa, and all strains identified as *P. delicatissima/P*. cf. *arenysensis* by the HRM assay grouped into the clade of *P. arenysensis*, which separated after *P. delicatissima*. Only three representative strains of Adriatic *P*. cf. *arenysensis* were shown. Hence, these two clades diverged after *P. micropora*. All the clades were supported by high bootstrap and posterior probability values (Figure S3).

## Discussion

In this study, a reliable, rapid and robust molecular qPCR HRM assay was developed in order to rapidly and accurately detect potentially harmful *Pseudo-nitzschia* species in cultured samples obtained from coastal water survey. This HRM method is based on a post PCR analysis, which differs from previous qPCR melt curve analyses^13, 30, 51^, because the amplicons produced by the qPCR are subjected to a thermal gradient with temperature increments of 0.1 °C/sec using sensitive instrumentation that ensures the absolute precision of the temperature increments. By continuously monitoring the fluorescence emitted by the Meltdoctor HRM Dye, it is possible to assess the exact melting temperature of the amplicon with a precision of 0.1 °C. Base differences and/or insertions or deletions of one or more bases are revealed, and this makes it possible to discriminate between amplicons and, consequently, between species. The genus specific primers were designed on a partial domain LSU rDNA sequence alignment including most representative *Pseudo-nitzschia* spp. species from the Mediterranean region in order to include a high level of genetic diversity. The variable D1–D3 region of LSU has been widely used for species-specific identification using various molecular approaches such as qPCR^13, 30^, microarray^32, 34, 52^, and FISH or sandwich hybridization assay^53–55^. Within the LSU gene, we identified a variable inter-specific target, flanked by highly conserved regions, which was suitable for primer design and the relative production of specific amplicons of each HRM variant. The ITS regions were also explored for primer design; however, they showed too much variability to encompass target *Pseudo-nizschia* species. In fact, ITS regions of diatom species are also known to be highly variable at intra-species level^56, 57^. The amplicon length was 130 bp satisfying the HRM analysis conditions. The sequence variations within the analyzed region of the LSU gene allowed us to use the HRM assay for the identification of these isolates. In particular, *P. delicatissima* and *P*. cf. *arenysensis* constituting the *P. delicatissima* complex, showed no differences in their nucleotide sequences and their high resolution melting curves were identical. Other molecular approaches, such as dot-blot hybridization^35^, have also shown that these species share identical target LSU regions because of incomplete lineage sorting^23^. When the *P. delicatissima* complex sequence was selected as a reference in the pairwise alignment analysis, *P. calliantha* and *P. pungens* showed 5 and 7 nucleotides of difference, respectively. These sequence features allowed us to distinguish these species and/or complex using the HRM assay. The melting curves of all the isolates could be clustered into three groups. Identification of the *Pseudo-nitzschia* spp. was made by comparing their values to those of controls, with a confidence interval of between 96–100% for auto-called results. The resulting melt profile reflected the difference in the amplicons and/or GC content in the 130 bp amplicons. The HRM assay was applied to *Pseudo-nitzschia* spp. unidentified isolates collected in the NW Adriatic Sea during a survey period. Distinct species were identified among the isolates, specifically *P. calliantha* (12 isolates), *P. pungens* (7 isolates) and *P. delicatissima*/*P*. cf. *arenysensis* (10 isolates). No cross-reactivity or melt curve overlapping among the various species-specific DNAs was obtained. The Tm values were significantly different among the three identified variants of the *Pseudo-nitzschia*. Phylogenetic analyses of the LSU and ITS-5.8S rDNA sequences of *Pseudo-nitzschia* spp. isolates identified by the HRM assay confirmed their species-specific taxonomical designation. The phylogenetic inference obtained from rDNA sequences was robust demonstrating that the distinct clades of *P. calliantha*, *P. pungens*, *P*. cf. *arenysensis* that were included in the *P. delicatissima* complex were supported by high bootstrap values and Bayesian inferences. *P. delicatissima* is a cryptic species complex, comprising different genetic lineages^20, 23, 58^, including *P. arenysensis*. Therefore, the HRM assay was able to identify the *P. delicatissima* complex without discriminating the species because of a lack of SNPs in the amplicon between toxic *P. delicatissima* and non-toxic *P. arenysensis*. However, it has already been proven that in the NW Adriatic Sea, *P. delicatissima*, as well as *P. arenysensis* strains are non- toxic or have low toxicity^13, 59^. In any case, further investigation is needed to better characterize the Adriatic *P*. cf. *arenysensis*. In fact, this phylogenetic clade was distinct from the *P. arenysensis* found in other Mediterranean areas, showing that the *P. delicatissima* complex still includes cryptic or semi-cryptic species.

This post PCR HRM analysis can be performed rapidly and with high processivity. Several cultured samples can be examined in one workday, unlike qPCR or sequence analyses, which are more time intensive. In addition, the HRM assay is less costly than microarrays, which require the development of probe devices, or qPCR analysis. The HRM assay depends on available reference genotypes, and it analyzes monoclonal cultures of the species under investigation. Hence, to account for the potential intra-specific genetic variability of the *Pseudo-nitzschia* spp., as many *Pseudo-nitzschia* DNA controls as possible will be developed, a broader range of *Pseudo-nitzschia* species diversity will be tested in the area under investigation. The HRM assay is likely to be feasible and applicable given that distinct melt curves of target *Pseudo-nitzchia* species have been obtained accurately based on different Tm and silhouettes of the curves for each species or complex.

In this area of the Mediterranean Sea, there is widespread high intensity aquaculture farming. Recent findings regarding DA toxin accumulation in shellfish and clonal strains found to produce DA highlight the necessity of developing methods able to accurately and rapidly determine how many species of *Pseudo-nitzschia* are present and which are actually producing DA. This information could be of great importance in the assessment of the potential risk of real toxic events in target coastal areas including the Adriatic one investigated in this study.

The post PCR HRM assay developed in this study appears to be a promising tool for simultaneous detection and identification of the *Pseudo-nitzschia* spp. coupled with analytical detection of DA. The assay offers several advantages: it is specific, reproducible and rapid when applied to several simultaneously processed cultured samples. Future sampling of the numerous potentially harmful species in the Mediterranean Sea could take advantage of the application range of the HRM assay.

## Electronic supplementary material

Supplementary Material
