# Supplementary material for: A high resolution melting method for the molecular identification of the potentially toxic diatom Pseudo-nitzschia spp. in the Mediterranean Sea
Source: Sci Rep. 2017 Jun 26;7:4259. doi: 10.1038/s41598-017-04245-z (PMC5484702; doi:10.1038/s41598-017-04245-z)
Supplement: Supplementary file 1 — Supplementary Material [file 41598_2017_4245_MOESM1_ESM.doc]

**Supplementary material**

**Scientific Reports**

**A high resolution melting method for the molecular identification of the potentially toxic diatom *Pseudo-nitzschia* spp. in the Mediterranean Sea**

Laura Pugliese^1^, Silvia Casabianca^1,2^, Federico Perini^1^, Francesca Andreoni^1^, Antonella Penna^1,2,3*^

^1^Department of Biomolecular Sciences, University of Urbino, Viale Trieste 296, 61121 Pesaro, Italy

^2^Conisma, Consorzio Interuniversitario per le Scienze del Mare, Pz. Flaminio 9, 00196 Rome, Italy

^3^CNR–Institute of Marine Sciences (ISMAR), Largo Fiera della Pesca 60125 Ancona, Italy

*corresponding author: email: antonella.penna@uniurb.it

**Table S1.** List of *Pseudo-nitzschia* spp. strains isolated from

the NW Adriatic Sea, at Pesaro, 500 m off the coast, used in

the HRM development assay.

| Species | Strain | Sampling date |
| --- | --- | --- |
| *P.* cf. *arenysensis* | CBA 160 | 10 February 2013 |
| *P. calliantha* | CBA 59 | 18 September 2009 |
| *P. calliantha* | CBA 62 | 18 September 2009 |
| *P. calliantha* | CBA 70 | 24 October 2009 |
| *P. calliantha* | CBA 71 | 24 October 2009 |
| *P. calliantha* | CBA 72 | 24 October 2009 |
| *P. calliantha* | CBA 73 | 24 October 2009 |
| *P. calliantha* | CBA 74 | 24 October 2009 |
| *P. delicatissima* | CBA 131 | 19 March 2010 |
| *P. delicatissima* | CBA 133 | 19 March 2010 |
| *P. delicatissima* | CBA 144 | 15 April 2010 |
| *P. delicatissima* | CBA 145 | 15 April 2010 |
| *P. delicatissima* | CBA 150 | 12 May 2010 |
| *P. delicatissima* | CBA 152 | 12 May 2010 |
| *P. delicatissima* | CBA 153 | 12 May 2010 |
| *P. pungens* | CBA 94 | 30 December 2009 |
| *P. pungens* | CBA 100 | 20 January 2010 |
| *P. pungens* | CBA 101 | 20 January 2010 |
| *P. pungens* | CBA 102 | 20 January 2010 |
| *P. pungens* | CBA 103 | 20 January 2010 |
| *P. pungens* | CBA 105 | 20 January 2010 |
| *P. pungens* | CBA 111 | 20 January 2010 |

**Table S2.** List of *Pseudo-nitzschia* spp. isolates, sampling location, LSU and ITS-5.8S gene sequence accession numbers. Isolates and sequence codes in bold are from this study.

| Species | Strain ID | Geographical origin | Accession no. LSU | Accession no. ITS |
| --- | --- | --- | --- | --- |
| *P. arenysensis* | 14V | Spain | AY764136 | AY764136 |
| *P. arenysensis* | 2b | California, USA | KT189137 | - |
| *P. arenysensis* | AL-24 | Naples, Italy, Tyrrhenian Sea | DQ813811 | - |
| *P. arenysensis* | AL-11 | Naples, Italy, Tyrrhenian Sea | - | DQ813840 |
| *P.* cf. *arenysensis* | **CBA159** | Pesaro, Italy, Adriatic Sea | **LT596179** | **LT596202** |
| *P.* cf. *arenysensis* | **CBA160** | Pesaro, Italy, Adriatic Sea | **LT596193** | - |
| *P.* cf. *arenysensis* | **CBA161** | Pesaro, Italy, Adriatic Sea | **LT596190** | - |
| *P.* cf. *arenysensis* | **CBA163** | Pesaro, Italy, Adriatic Sea | **LT596180** | **LT596194** |
| *P.* cf. *arenysensis* | **CBA165** | Pesaro, Italy, Adriatic Sea | **LT596181** | - |
| *P.* cf. *arenysensis* | **CBA166** | Pesaro, Italy, Adriatic Sea | **LT596200** | - |
| *P.* cf. *arenysensis* | **CBA167** | Pesaro, Italy, Adriatic Sea | **LT596192** | **LT596195** |
| *P.* cf. *arenysensis* | **CBA168** | Pesaro, Italy, Adriatic Sea | **LT596201** | - |
| *P.* cf. *arenysensis* | **CBA169** | Pesaro, Italy, Adriatic Sea | **LT596182** | - |
| *P.* cf. *arenysensis* | **CBA170** | Pesaro, Italy, Adriatic Sea | **LT596189** | - |
| *P.* cf. *arenysensis* | **CBA171** | Pesaro, Italy, Adriatic Sea | **LT596191** | - |
| *P. arenysensis* | ICMB130 | Barcelona, Spain | - | EU367952 |
| *P. caciantha* | AL-56 | Naples, Italy, Tyrrhenian Sea | DQ813812 | DQ813834 |
| *P. calliantha* | AL-112 | Naples, Italy, Tyrrhenian Sea | DQ813815 | DQ813841 |
| *P. calliantha* | CBA62 | Pesaro, Italy, Adriatic Sea | **LT596176** | HE663423 |
| *P. calliantha* | CBA72 | Pesaro, Italy, Adriatic Sea | **LT596175** | HE663433 |
| *P.* *calliantha* | CBA192 | Pesaro, Italy, Adriatic Sea | **LT596184** | - |
| *P.* *calliantha* | CBA193 | Pesaro, Italy, Adriatic Sea | - | **LT596205** |
| *P. calliantha* | CBA194 | Pesaro, Italy, Adriatic Sea | **LT596183** | **LT596206** |
| *P. cuspidata* | AL-17 | Naples, Italy, Tyrrhenian Sea | DQ 813809 | DQ813827 |
| *P. cuspidata* | PA7 | Sydney, Australia | KC017453 | - |
| *P. delicatissima* | 1001 2 B | Copenhagen, Denmark | AF417645 | - |
| *P. delicatissima* | ICMB134 | Tarragona, Spain | - | EU327383 |
| *P. delicatissima* | AL 22 | Naples, Italy, Tyrrhenian Sea | DQ813810 | DQ813832 |
| *P. delicatissima* | AY4 | Gauteng, South Africa | EF522107 | - |
| *P. delicatissima* | CBA144 | Pesaro, Italy, Adriatic Sea | **LT596187** | HE650934 |
| *P. delicatissima* | CBA145 | Pesaro, Italy, Adriatic Sea | **LT596188** | HE650935 |
| *P. delicatissima* | CLA1.A1 | Gauteng, South Africa | EF522114 | - |
| *P. dolorosa* | AL-59 | Naples, Italy, Tyrrhenian Sea | DQ813813 | DQ813835 |
| *P. fraudulenta* | LIMENS1 | Copenhagen, Denmark | AF 417647 | AY257840 |
| *P. fukuyoi* | PnTb72 | Sarawak, Malaysia | KC147537 | - |
| *P. hasleana* | HAWK3/1 | Sydney, Australia | KC 017446 | - |
| *P. hasleana* | HAWK4 | Sydney, Australia | - | KC017468 |
| *P. hasleana* | NWFSC186 | Copenhagen, Denmark | JN050298 | - |
| *P. inflatula* | No7 | Copenhagen, Denmark | - | DQ329204 |
| *P. mannii* | AL-101 | Naples, Italy, Tyrrhenian Sea | DQ813814 | DQ813839 |
| *P. micropora* | VPB-B3 | Copenhagen, Denmark | AF 417649 | AY257847 |
| *P. multiseries* | OFPm984 | Copenhagen, Denmark | AF417655 | DQ062664 |
| *P.* *multistriata* | **CBA174** | Pesaro, Italy, Adriatic Sea | **LT596185** | **LT596196** |
| *P. multistriata* | PSM11 | Primorsky krai, Russian Federation | - | KT247444 |
| *P. multistriata* | SZN-B31 | Naples, Italy, Tyrrhenian Sea | AF416756 | - |
| *P. multistriata* | SZN-B32 | Naples, Italy, Tyrrhenian Sea | AF416757 | - |
| *P. pseudodelicatissima* | 8A 14 | Thermaikos Gulf, Greece | FJ859054 | - |
| *P. pseudodelicatissima* | 9A 1 | Thermaikos Gulf, Greece | FJ859055 | - |
| *P. pseudodelicatissima* | AL-15 | Naples, Italy, Tyrrhenian Sea | DQ 813808 | DQ813826 |
| *P. pseudodelicatissima* | **CBA175** | Pesaro, Italy, Adriatic Sea | **LT596186** | - |
| *P. pseudodelicatissima* | P11 | Helsingor, Denmark | AF 417640 | AY257854 |
| *P. pseudodelicatissima* | SZN-B545 | Naples, Italy, Tyrrhenian Sea | KF241716 | - |
| *P. pungens* | CBA100 | Pesaro, Italy, Adriatic Sea | **LT596177** | HE650958 |
| *P. pungens* | CBA111 | Pesaro, Italy, Adriatic Sea | **LT596178** | HE650968 |
| *P. pungens* | CBA179 | Pesaro, Italy, Adriatic Sea | **LT596197** | **LT596203** |
| *P. pungens* | CBA180 | Pesaro, Italy, Adriatic Sea | **LT596198** | **LT596204** |
| *P. pungens* | CBA182 | Pesaro, Italy, Adriatic Sea | **LT596199** | - |


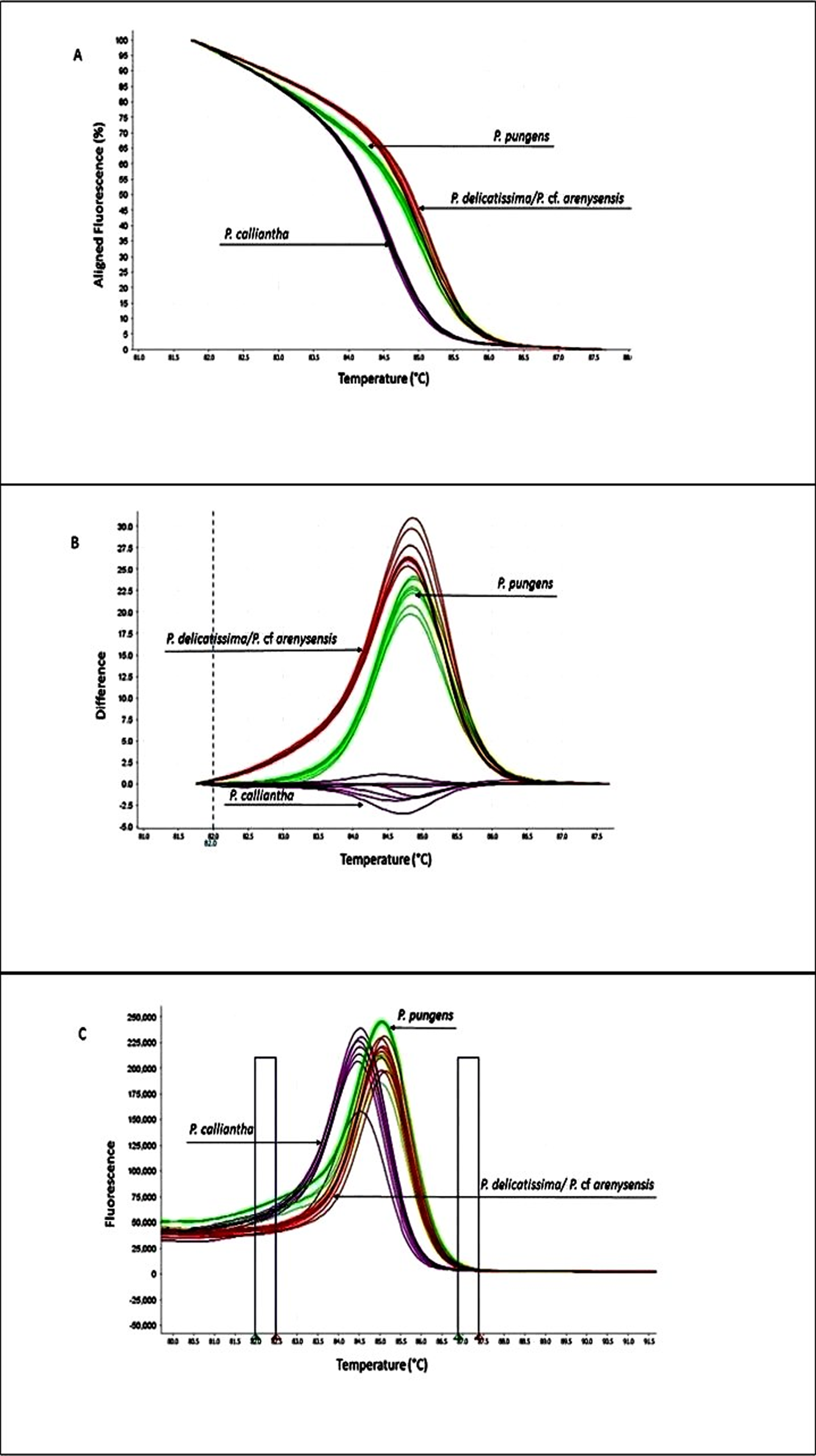


Figure S1. Melting curve variance of the three *Pseudo-nitzschia* species controls (*P. calliantha,* n = 7 strains, *P. delicatissima/P. cf. arenysensis,* n = 8 strains, *P. pungens* n = 7 strains) in (A) aligned, (B) difference and (C) derivative plot analyses; from left to right vertical bars represent the pre and post – melt regions. Only one replicate of the HRM assay experiment for each strain is shown.

Fig. S2. Maximum likelihood phylogenetic tree of the genus *Pseudo-nitzschia* inferred from LSU rDNA. The tree was rooted with *Fragilariopsis* *rhombica* 5-17 as outgroup. Numbers of the major nodes represented from left to right or from upper to lower NJ (1000 pseudo-replicates), MP (1000 pseudo-replicates), ML (1000 pseudo-replicates) bootstrap and Bayesian posterior probability values. Only bootstrap values > 50% were shown. All sequences of bold isolates were obtained in this study.

Fig. S3. Maximum likelihood phylogenetic tree of the genus *Pseudo-nitzschia* inferred from ITS-5.8S rDNA. The tree was rooted with *Fragilariopsis* sp. NL2010 as outgroup. Numbers of the major nodes represented from left to right NJ (1000 pseudo-replicates), MP (1000 pseudo-replicates), ML (1000 pseudo-replicates) bootstrap and Bayesian posterior probability values. Only bootstrap values > 50% were shown. All sequences of bold isolates were obtained in this study.
